# Clinical Evidence for the Choice of the Direct Oral Anticoagulant in Patients with Atrial Fibrillation According to Creatinine Clearance

**DOI:** 10.3390/ph14030279

**Published:** 2021-03-19

**Authors:** Riccardo Vio, Riccardo Proietti, Matteo Rigato, Lorenzo Arcangelo Calò

**Affiliations:** 1 Department of Cardiac, Thoracic, and Vascular Sciences and Public Health, University of Padova, 35128 Padova, Italy; riccardo.vio.1@gmail.com (R.V.); riccardoproietti6@gmail.com (R.P.); 2 Department of Medicine, Nephrology, Dialysis and Transplantation Unit, University of Padova, 35128 Padova, Italy; matteo.rigato@hotmail.it

**Keywords:** direct oral anticoagulants, chronic kidney disease, atrial fibrillation, stroke, bleeding

## Abstract

Atrial fibrillation (AF) often coexists with chronic kidney disease (CKD), which confer to the patient a higher risk of both thromboembolic and hemorrhagic events. Oral anticoagulation therapy, nowadays preferably with direct oral anticoagulants (DOACs), represents the cornerstone for ischemic stroke prevention in high-risk patients. However, all four available DOACs (dabigatran, apixaban, rivaroxaban and edoxaban) are eliminated by the kidneys to some extent. Reduced kidney function facilitates DOACs accumulation and, therefore, different dose reductions are required, with slight differences between American and European recommendations especially in case of severe renal impairment (creatinine clearance < 30 mL/min). Overall, the use of DOACs in patients with non-end stage CKD and AF is effective similarly to warfarin, showing a better safety profile. The management of thromboembolic risk among patients with AF on dialysis remains challenging, as warfarin effectiveness for stroke prevention in this population is questionable and retrospective data on apixaban need to be confirmed on a larger scale. In kidney transplant recipients, DOACs may provide a potentially safer option compared to warfarin, but co-administration with immunosuppressants is a matter of concern.

## 1. Introduction

Atrial fibrillation (AF) and chronic kidney disease (CKD) are on the rise worldwide. Both disorders are strictly related to ageing population and may coexist in the same patient. CKD affects 15% of adults, who in turn will suffer from AF in up to 30% of cases depending on the severity of the renal impairment [1,2]. The factors predisposing to AF in patients with CKD include hypertension, heart failure and autonomic imbalance, leading to structural and electrical remodeling of the atria [3]. Once appeared, AF favors CKD progression resulting in a vicious cycle [4,5]. Thromboembolic complications typical of AF are amplified in patients with renal dysfunction, who may have a hypercoagulable state due to increased platelet activity, activation of the renin-angiotensin-aldosterone system, altered vessel wall contractility and vascular endothelium changes, increasing the risk of stroke by 50% [4,6,7,8]. 

Oral anticoagulation therapy, nowadays preferably with direct oral anticoagulants (DOACs), represents the cornerstone for stroke thromboprophylaxis in high-risk patients with AF according to CHA2DS2VASc score [9,10]. The impaired renal function which defines CKD directly impacts on the anticoagulation regimen, since all four available DOACs are eliminated at least partially by the kidneys. A correct prescription is fundamental because adverse events due to supratherapeutic levels include major bleeding such as hemorrhagic stroke. These unfavorable events are enhanced in patients with CKD, since the alteration of the hemostatic system may include also a hemorrhagic diathesis led by platelet dysfunction, compromised platelet aggregation and intercurrent anemia [11]. 

**Figure 1:**
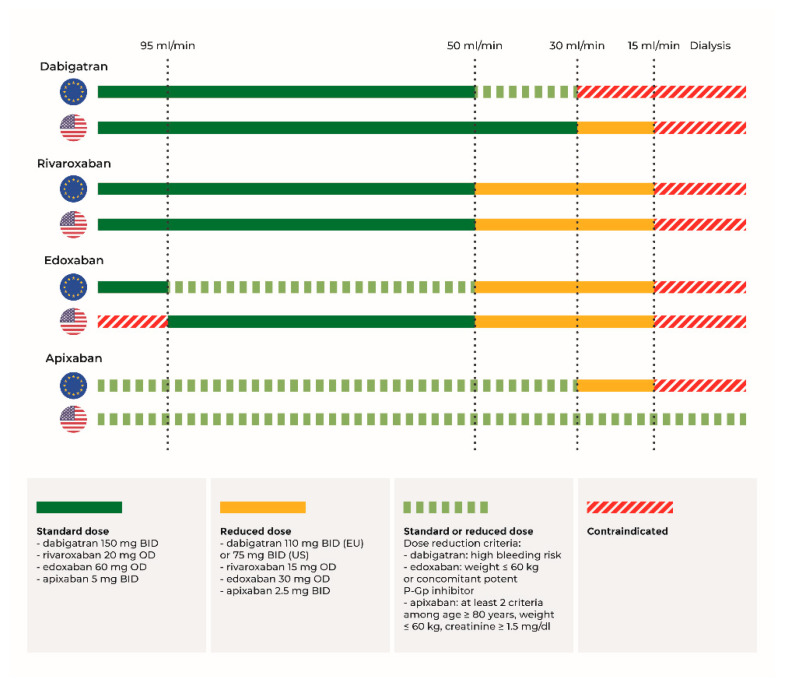
Recommendations for direct oral anticoagulants dosing on the basis of renal function according to European and American guidelines.

In this review we discuss the current evidence regarding efficacy and safety profiles of DOACs according to different clinical setting (non-end-stage and end-stage CKD). International recommendations (European and American) for DOACs dosing are presented thought the text and reassumed in Figure 1.

European and American recommendations are based on the latest 2018 EHRA and 2019 AHA/ACC/HRS documents [12,13]. BID = bis in die; OD = omne in die; EU = Europe; US = United States; P-Gp = P-glycoprotein. 

## 2. Non-End-Stage Chronic Kidney Disease

In the past decades, warfarin was the preferred anticoagulant used for stroke prevention in patients with AF. The limitations related to warfarin therapy (i.e., slow onset of action, variable pharmacologic effects, several food and drug interactions) were overcome by the advent of DOACs, that in turn demonstrated a similar efficacy and better safety profile. All DOACs show a predictable pharmacokinetic and do not require regular monitoring of the coagulation to optimize their clinical management. Warfarin is metabolized by the liver and so it is routinely used even in patients with end-stage CKD. Conversely, DOACs are eliminated by the kidneys to some extent and their use in patients with impaired renal function raises some concerns. Dabigatran has the highest renal elimination (80%), whereas edoxaban, rivaroxaban and apixaban have lower values (50%, 35% and 27%, respectively) [12]. The Cockcroft-Gault formula is commonly used to calculate, on the basis of serum creatinine levels, the creatinine clearance (CrCl, expressed in ml/min), that is an estimation of the glomerular filtration rate (GFR). Other equations express the GFR in ml/min/1.73m^2^, such as CKD-EPI, which is used to classify the severity of CKD [14]. For the sake of dosing the DOACs, CrCl calculated with Cockcroft-Gault formula should be used as a reference, since it was adopted by all the following four randomized controlled trials [15,16,17,18]. The RE-LY, ROCKET AF and ENGAGE AF-TIMI 48 trials, respectively, tested efficacy and safety of dabigatran, rivaroxaban and edoxaban and excluded patients with severe CKD (i.e., CrCl <30 mL/min) [15,16,18]. The ARISTOTELE trial instead studied apixaban and included patients with CrCl up to 25 mL/min, since this DOAC has the lowest renal elimination [17]. Numerous post-hoc analysis of these trials investigated whether or not DOACs efficacy and safety were confirmed in patients with mild to moderate CKD compared to those with normal renal function. 

In the study by Hijazi et al. the efficacy and safety of dabigatran compared to warfarin were analyzed in relation to baseline renal function [19]. After estimating the GFR with the CKD-EPI formula, instead of the Cockcroft-Gault equation used in the trial, he found significant interactions between treatment and renal function: both dabigatran dosages (150 and 110 mg bis in die (BID)) displayed lower rates of major bleeding in patients with GFR >80 mL/min, while their efficacy was consistent with the overall trial regardless of renal function [19]. In patients with severe renal dysfunction (GFR 15–29 mL/min), pharmacological projections suggested a reduced dose of dabigatran 75 mg BID: US Food and Drug Administration allows the administration of such regimen in this case, but dabigatran remain contraindicated in Europe when GFR is < 30 mL/min because of safety concerns [12,13,20]. 

For rivaroxaban, other pharmacokinetics analysis demonstrated that maximal serum concentrations were 25–30% higher in patients with moderate CKD (CrCl 30–49 mL/min) [21]. This evidence triggered, in the ROCKET AF trial, the reduction of rivaroxaban dosage from 20 mg omne in die (OD) to 15 mg OD in patients with moderate renal insufficiency, who represented a sizeable proportion of the study population (one in five study patients) [16]. The overall study demonstrated a benefit in stroke reduction and systemic embolism comparable to warfarin, with fewer fatal hemorrhages. A post-hoc analysis demonstrated that rivaroxaban 15 mg in moderate renal impairment yielded the same results of the standard 20 mg in preserved kidney function, excluding a heterogeneity in treatment effect across dosing groups [22]. The administration of rivaroxaban 15 mg OD is permitted both in Europe and in US even in case of severe renal impairment (GFR 15–29 mL/min), despite this condition represented an exclusion criterion for the trial [12,13]. 

Another DOAC approved in case of GFR > 15 mL/min is edoxaban [12,13]. The clearance of edoxaban depends for 50% on renal elimination, and total drug exposure increases by 32 to 72% in patients with mild to severe renal dysfunction [23]. Therefore, in the ENGAGE AF-TIMI 48 trial the edoxaban dose was reduced from 60 to 30 mg OD in patients with moderate renal insufficiency (CrCl 30–49 mL/min); patients with severe reduction of renal function (CrCl < 30 mL/min) were excluded [18]. Other criteria for dose reduction were weight ≤ 60 kg or administration of a strong P-glycoprotein inhibitor. Compared to warfarin, edoxaban had a comparable efficacy for stroke prevention, carrying a reduced risk for bleeding and cardiovascular death [18]. Bleeding rates were lower at all levels of CrCl, as later assessed by Bohula et al. [24]. A further post-hoc analysis added useful evidence that in patients who developed severe chronic impairment during follow-up (CrCl < 30 mL/min) stroke and major bleeding rates were similar between those treated with edoxaban compared to those on warfarin [25]. On the other hand, edoxaban 60 mg may be less effective for stroke prevention in patients with “supranormal” renal function (CrCl > 95 mL/min) and in such cases it is contraindicated in the US, whereas European recommendations indicate a cautionary use [12,13,18,24]. 

Apixaban has the minimal renal excretion compared to the other DOACs (only 27%) [12], and it is commercialized in two dosages, 5 or 2.5 mg BID. Criteria for dose reduction include not only creatinine values (≥ 1.5 mg/dl), but also weight ≤ 60 kg and age ≥ 80 years: when at least two out of the three criteria are met, 2.5mg BID should be prescribed [12,13]. Hohnloser et al. analyzed data of the ARISTOTELE trial and found that apixaban compared to warfarin reduced the rate of stroke, death and major bleeding irrespective of renal function [26]. Of note, the patients who benefited the most from reduction of serious bleeding events where those having at least a moderate renal impairment (CrCl < 50 mL/min). Corroborative evidence was provided by a recent post-hoc analysis focusing on the enrolled patients with the lowest CrCl (25–30 mL/min): in such patients with severe renal impairment apixaban treatment was associated with a greater reduction in bleedings compared to those having a CrCl > 30 mL/min [27]. In severe CKD (CrCl 15–30 mL/min), apixaban is approved in US with the same aforementioned criteria for moderate renal impairment, whereas in Europe it is permitted at the lowest dosage (2.5 mg BID) irrespective of age and weight [12,13].

For all DOACs, regular calculation of CrCl during follow-up is required to check for a possible decline of renal function. CKD is a progressive disease and incident AF has been independently associated with a 67% higher rate of progression to the end-stage phase [4,5]. European experts recommend monitoring renal function more than once per year when baseline CrCl is reduced (< 60 mL/min). The proposed formula for calculating the rechecking interval in months of the renal function during DOAC therapy is CrCl/10 (e.g., for CrCl 40 mL/min the rechecking interval is four months) [12]. Periodic reassessment of renal function helps to timely identify CrCl decline, avoiding DOACs accumulation at supratherapeutic plasma levels that may cause major bleedings.

## 3. End-Stage Chronic Kidney Disease

End-stage renal disease is classified under stage 5 of CKD, together with persons with a GFR < 15 mL/min/1.73 m^2^ [14]. It is defined as permanent loss of kidney function, that invariably leads to death unless dialysis or transplantation are pursued. Herein are described evidence and current practice regarding oral anticoagulation in these two different clinical scenarios.

## 4. Patients on Dialysis

Patients with CKD on dialysis have almost a double risk of stroke compared to non-end-stage counterparts [28]. In addition, all nephropathic patients show some degree of platelet disfunction and impaired platelet aggregation, that are even more pronounced in end-stage CKD; the so-called “uremic platelet dysfunction” further augments the bleeding risk [29,30]. 

In this clinical scenario, the side-effects of anticoagulation therapy might offset the desired benefit. Available evidence showed that warfarin increased the rate of bleeding events without any impact on stroke prevention or death [31]. A recent meta-analysis gathered all the literature in the field and confirmed warfarin inefficacy for ischemic stroke prevention among patients on dialysis; hemorrhagic strokes were significantly higher in those receiving treatment, but mortality was comparable between groups [32]. Factors that can explain these observations include the routine administration of heparin during dialysis and the interference of uremic state with the metabolisms of warfarin, making it difficult to maintain the international normalized ratio in the therapeutic range [33,34,35,36]. Apart from growing concern on the trade-off between harm and benefit, warfarin seems to accelerate the worsening of renal function either by favoring parenchymal microbleeds or by promoting vascular calcifications [37,38]. All the above-mentioned considerations raised the urgent need for new therapeutic approaches, looking at DOACs as appealing alternatives to warfarin in this medically complex population. 

European recommendations contraindicate the use of all DOACs in patients with CrCl < 15 mL/min or on dialysis, whereas US Food and Drug Administration allows only apixaban in these cases [12,13]. Following this labelling, the use of apixaban has grown in American patients with AF on dialysis, accounting for approximately one out of four new anticoagulants prescribed in this population [39]. In a recent study, Siontis and colleagues retrospectively compared the rate of stroke/systemic embolism, major bleedings and death between patients on dialysis treated with apixaban versus warfarin [39]. The researchers concluded that both standard and reduced dose of apixaban (5 and 2.5 mg BID, respectively) were associated with a lower risk of major bleedings, but only the standard dose significantly reduced thromboembolic events and death compared to warfarin. However, they described also high rates of intracerebral bleedings and drug discontinuations, casting doubts on the real progress in the management of these complex patients. 

Despite being contraindicated, Chan et al. found that prescription of dabigatran and rivaroxaban was occurring among patients on dialysis in US [40]. The authors reported that either drugs were associated with a higher rate of hospitalization for bleedings and hemorrhagic death, supporting the current labeling of dabigatran and rivaroxaban. More recent evidence showed that a reduced dose of rivaroxaban (10 mg OD) may lower severe bleeding complications compared to warfarin in hemodialysis patients [41]. 

New randomized clinical trials are under way and will shed some lights on the outcome of DOACs for stroke prevention in patients with AF on hemodialysis. The RENAL-AF trial (Renal Hemodialysis Patients Allocated Apixaban Versus Warfarin in Atrial Fibrillation trial; ClinicalTrials.gov (accessed on 14 February 2021) identifier NCT02942407) was prematurely stopped for failure to enroll a sufficient number of patients [42]. Other ongoing studies are similarly attempting to compare the efficacy and safety of apixaban versus vitamin-K antagonists for stroke prevention in patients with AF and end-stage CKD. These studies include the SAFE-HD trial (Strategies for the Management of Atrial Fibrillation in Patients Receiving Hemodialysis; ClinicalTrials.gov (accessed on 14 February 2021) identifier NCT03987711) and the AXADIA trial (Compare Apixaban and Vitamin-K Antagonists in Patients with Atrial Fibrillation and End-Stage Kidney Disease; ClinicalTrials.gov (accessed on 14 February 2021) identifier NCT02933697). The design of the SAFE-HD trial provided for a control arm of patients without anticoagulation, since previous studies pointed out the ineffectiveness and potential harm of oral anticoagulation with warfarin in patients receiving dialysis [32]. For now, the relatively scarce amount of data supporting the use of apixaban in patients with severe renal impairment warrant caution [43], but the sheer volume of patients recruited in the abovementioned trials will hopefully allow to completely assess the topic.

Left atrial appendage occlusion (LAAO) represents a nonpharmacological alternative for stroke prevention in AF patients with end-stage renal disease or on hemodialysis [44]. A recent subanalysis of the LAARGE registry revealed that LAAO was safe and associated with effective stroke prevention across all CKD stages, including stage 5 (GFR < 15 mL/min/1.73 m^2^) [45]. The available studies aimed to compare LAAO with anticoagulant therapy showed a non-inferiority of LAAO compared to warfarin [46,47]. However, since they excluded patients treated with DOACs, non-inferiority of LAAO against DOACs cannot be assumed.

## 5. Kidney Transplant Recipients

AF occurs in over 7% of kidney transplant recipients in the first three years after transplantation and is higher in the peri-transplant period [48]. New onset of AF in kidney transplant recipients confers a worse prognosis and is associated with a reduced graft and patient survival [48]. In high-risk patients according to CHA2DS2VASc score, the start of oral anticoagulation therapy is indicated [9]. Since recent years, vitamin k antagonists have been the only oral anticoagulant agents available. An analysis performed on a US registry of patients with end-stage CKD failed to show a significant reduction of a composite endpoint of mortality and stroke in patients who underwent renal transplant and were treated with warfarin for new onset AF [49]. Accordingly, the authors reported that, in the overall population with AF and renal transplant, warfarin was under-prescribed, possibly due to lack of evidence for a clinical benefit and a perceived increased risk of bleeding in hemodialyzed patients [49]. 

DOACs provide a potentially safer option in patients who have had renal transplant. However, they have at least a partial renal excretion and so exposure can increase in patients with CKD, including those who received kidney transplant. Ischemia-induced injury to the kidney both during the procurement period and transplant surgery can lead to temporary reduced graft function in the immediate post-transplant period, with delayed or slow graft function that require hemodialysis in 25% of deceased-donor recipient and 3–5% of living donor recipient [50]. At the moment, no clinical trial data is available for DOACs use in patients post renal transplant, a population with a unique challenge: maintenance of an effective immunosuppression. 

Tacrolimus and ciclosporin are two calcineurin inhibitors and commonly used immunosuppressants. Both have a narrow therapeutic index and blood concentrations vary considerably between individuals. In transplant recipients, both supratherapeutic and subtherapeutic drug concentrations can have devastating results. Subtherapeutic levels increase the risk of transplant rejection and supratherapeutic levels (over-immunosuppression) can lead to infection and/or drug-specific side effects [49]. Vanhove et al. assessed the effect of DOACs on the disposition of calcineurin inhibitors in patients who underwent renal transplant [51]. The study included 39 kidney recipients (29 on rivaroxaban and 10 on apixaban) and found only a slight and not clinically significant increase in calcineurin inhibitors concentration. A recent statement of European Heart Association on the basis of known metabolic pathways of calcineurin inhibitors (CYP3A and efflux pump P-glycoprotein), suggests that apixaban may be used in association with tacrolimus and cyclosporin with close monitoring and dose adjustment [12]. More recently, a study of drug interaction between apixaban and calcineurin inhibitors in healthy volunteers showed normal fluctuations of drug levels [52]. Therefore, no dose adjustment of the drug was needed during co-administration of apixaban with calcineurin inhibitors in healthy volunteers [52]. 

## 6. Conclusions

In summary, the use of DOACs in patients with non-end stage CKD and AF is effective for ischemic stroke prevention similarly to warfarin, showing an overall better safety profile. European and American recommendations slightly differ with regard to DOACs labeling, particularly in case of severe renal impairment. The observational data regarding the use of warfarin in AF patients on dialysis warrant caution; some retrospective data demonstrated promising results with apixaban, which need to be confirmed on a larger scale. Patients who underwent kidney transplant may benefit as well from the use of DOACs, but possible interactions with lifesaving immunosuppressants raise some concerns.

## Data Availability

No new data were created or analyzed in this study. Data sharing is not applicable to this article.
